# Intracranial Infantile Hemangioma: Highlighting a Rare Presentation With a Case Report and Literature Review

**DOI:** 10.7759/cureus.52341

**Published:** 2024-01-15

**Authors:** Sultan F Albalawi, Badr E Hafiz, Alanoud Turki, Murad Alturkustani, Salwa I Bakhsh, Abdulrahman J Sabbagh

**Affiliations:** 1 Neurological Surgery, King Abdulaziz University Hospital, Jeddah, SAU; 2 Neurosurgery, King Faisal Specialist Hospital and Research Centre, Jeddah, SAU; 3 Neurology, King Abdulaziz University Hospital, Jeddah, SAU; 4 Medicine, King Abdulaziz University Faculty of Medicine, Jeddah, SAU; 5 Pathology, King Abdulaziz University Hospital, Jeddah, SAU; 6 Training Administration, Saudi Commission for Health Specialties, Makkah, Jeddah and Taif, SAU; 7 Surgery, King Abdulaziz University Hospital, Jeddah, SAU

**Keywords:** neuropathology, cd31, histopathology, surgical resection, case report, intracranial infantile hemangioma

## Abstract

Infantile hemangioma is a common benign vascular tumor in children, but it is very unusual to be found intracranially. Our literature review identified 44 reported cases. Presentation can vary from asymptomatic to a life-threatening presentation that necessitates urgent surgical removal. There is no general consensus on management of these rare lesions and until recently, treatment was limited to surgery or pharmacological management with steroids, propranolol or interferon. We present a case of a four-week-old male infant with history of vomiting and increase in head circumference since birth. MRI of the brain revealed a large complex cyst occupying the right frontoparietal region, with round soft tissue component that is isointense on T1 and hyperintense on T2 weighted images. Complete surgical resection with evacuation of the cyst was achieved. Histopathology of the mass showed infantile hemangioma with positive CD31 on immunohistochemistry. The patient achieved an excellent outcome following surgical resection.

## Introduction

Infantile hemangioma is the most commonly encountered benign vascular tumor of childhood [1]. They typically tend to occur in superficial layers of the body more than deep layers. About 83% of these lesions affect the head and neck areas, but other parts of the body can be involved [2]. Intracranial involvement is very rare with incidence of 0.1%, especially when it is isolated from other organ involvement, with only scattered reports in literature [1,2]. Most documented cases were located in the cerebellopontine angle, with fewer reports describing other intracranial locations.

Hemangiomas have the capacity of rapid growth secondary to the rapid proliferative phase in the first 12 months of life [3]. The first five months of life are considered the peak for hemangioma growth, at which 80% of the final size has often reached, reaching the maximal size by nine months [3,4]. This period is followed by a gradual involutional phase, in which 90% of hemangiomas have spontaneously involuted by the age of five years [3,4]. Treatment is not required for hemangiomas in general. However, intracranial hemangiomas require intervention as the rapid growth may result in severe neurological complications. Different modalities were used to treat intracranial infantile hemangiomas, though literature is lacking solid information on standard treatment [4].

We performed a literature review using Google Scholar and Pubmed searching for keywords “intracranial infantile hemangioma/s”, and identified 44 reported cases [5-7]. Cases with asymptomatic small hemangiomas were managed with watchful waiting. Most of the cases were treated medically with oral prednisolone, oral propranolol, and intralesional triamcinolone, interferon alpha or thalidomide. However, only nine cases were treated surgically [5-7]. The efficacy of these measures was variable, with significant side effects noted in some cases [5-7].

This article was previously presented as a poster presentation at the 17th Saudi Association of Neurological Surgery conference on March 6, 2023. 

## Case presentation

**Figure 1:**
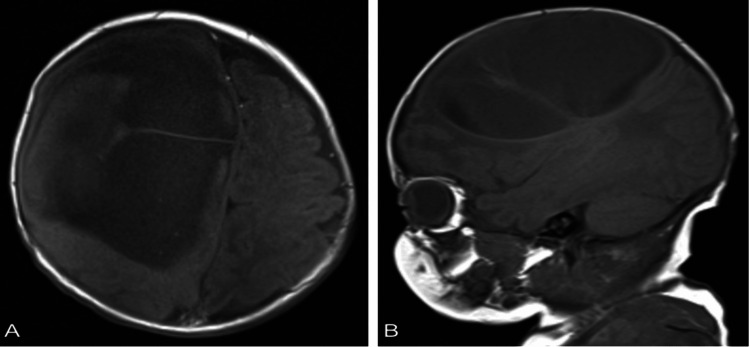
(A) axial T1-weighted MRI (B) sagittal T1-weighted MRI demonstrate a large intraparenchymal cystic mass with low signal intensity, located in the right frontoparietal region of the right cerebral hemisphere.

**Figure 2:**
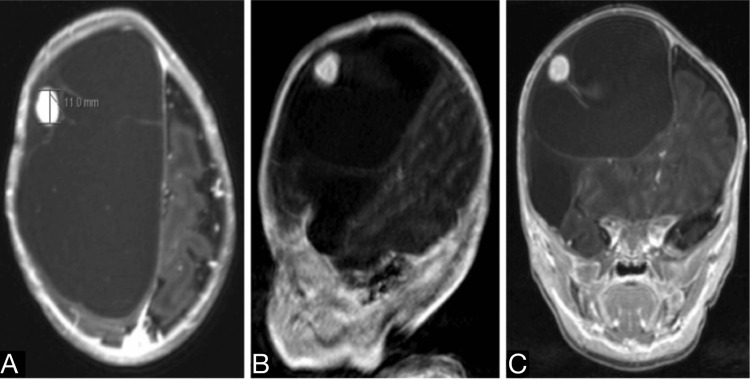
(A) Magnetization prepared rapid acquisition gradient echoes (MPRAGE) axial T1-weighted contrast-enhanced MRI, (B) sagittal T1-weighted contrast-enhanced MRI, (C) coronal T1-weighted contrast-enhanced MRI demonstrate a well-defined oval-shaped lesion that is homogeneously enhancing located at the lateral aspect of the mass.

This 30-day-old infant boy was referred to the Neurosurgery clinic with a space-occupying lesion demonstrated by neuroimaging. This boy presented since birth with vomiting and increased head circumference. His initial brain CT showed a right intra-axial frontal lesion with mural nodule and a significant mass effect. A detailed history revealed he is otherwise a healthy full-term neonate with no additional complaints. Full physical exam showed a head circumference above the 95th percentile, asymmetric facial appearance with a bulge of the right frontoparietal area, and wide bulging anterior fontanel. No skin lesions were present or other associated anomalies. A more extensive neuroimaging modality was requested. Gadolinium-enhanced magnetic resonance imaging (MRI) of the brain revealed a large 9.7 X 6.6 X 5.7 cm complex cyst occupying the right frontoparietal region, with a small round soft tissue component around 1 cm in diameter. It is isointense on T1 and hyperintense on T2 weighted images. Minimal micro bleed is identified within the septation (Figures 1, 2).

Based on the images, the provisional diagnosis was a desmoplastic infantile ganglioglioma.

**Figure 3:**
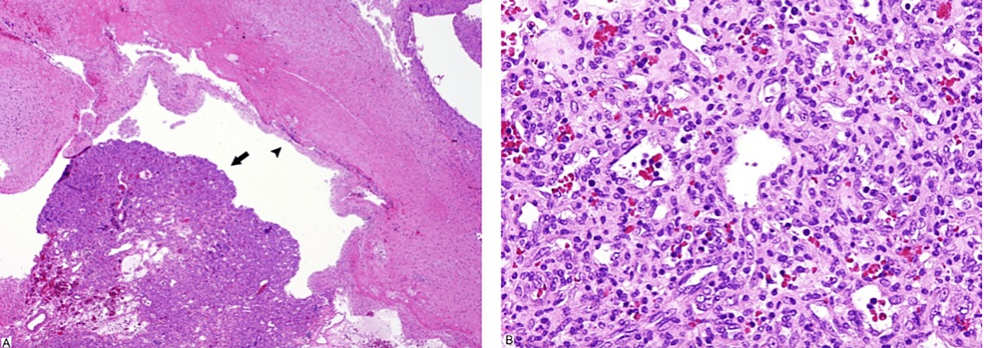
(A) The nodule in the cyst is formed by vascular neoplasm (arrow). The cyst wall (arrow’s head) is formed by reactive glial tissue. (B) The neoplasm is formed by cellular, and back-to-back capillary-size vascular spaces. Plumb endothelial cells with no atypia line these spaces. Haematoxylin & Eosin stain.

Management plan was conducted based on symptoms, huge mass size, significant compression of brain structures and resection of a suspicious brain tumor. Decompression of the intracranial tumor via osteoplastic craniotomy was done. Right fronto-parietal scalp incision and Mayo scissors were used to create the bone flap as the skull had been thinned out by the prolonged applied pressure. 50cc of brownish fluid was aspirated and the complete resection of the solid component was performed. Frozen section of the cystic wall was sent and showed normal brain parenchyma. Histopathology confirmed the diagnosis of infantile hemangioma with positive CD31 on immunohistochemistry (Figure 3). 

**Figure 4:**
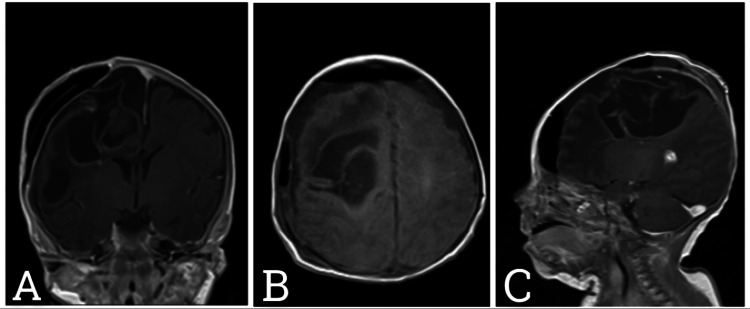
Postoperative T1-weighted MRI brain Coronal (A) Axial (B) Sagittal (C) views shows significant decompression of the large right cerebral cystic mass with almost no mass effects identified in the current MRI examination and no midline shift with normal configuration of the supratentorial and infratentorial ventricular system. Resection of the previously visualized well-defined rounded solid mass with no residual identified in the current MRI examination. Multiple hemorrhagic spots, pneumocephalus and mild subdural fluid are identified related to operative interference.

The surgery was uneventful without any intraoperative or postoperative complications. MRI brain postoperatively was done and showed complete resection of the solid portion of the tumor with no residual and partial decompression of the large cystic component with resolution of the mass effect (Figure 4).

The patient's vomiting ceased and he was stable postoperatively. He was discharged from the hospital within the first week after surgery. Upon clinic follow-up the head circumference was markedly reduced and he was doing well without any recurrent or new signs or symptoms. 

## Discussion

On patient admission in the described case, we considered a desmoplastic infantile ganglioglioma or a pilocytic astrocytoma as possible etiologies. The isolated intracranial involvement in our patient has precluded the possibility of infantile hemangioma. The occurrence in the frontoparietal region in our case is the first to be reported. 

To our knowledge, there are only 44 cases published in the literature (Table 1) and our case presented here is the 45th. Most of the cases were females (66.7%). The age at presentation ranges from 0-36 months with a mean of 3.7 months. The presenting symptoms were variable from case to case. 

**Table 1:** Cases of intracranial infantile hemangiomas CPA: cerebellopontine angle; IAC: internal auditory canal; CN: cranial nerve; VP: ventriculoperitoneal; ICP: intracranial pressure; PHACES: posterior fossa brain malformations, hemangiomas, arterial anomalies, coarctation of the aorta and cardiac defects, and eye abnormalities

Author, Year	Age at Presentation, Gender	Neurological or Ophthalmic Complaint	Intracranial Location of Hemangioma	Treatment	Outcome
Willing et al., 1993 [12]	17 months, male	Focal seizures, mild developmental delay	Right temporal dura	Surgical excision	Resolution
Bar-Sever et al., 1994 [13]	2 weeks, female	Nil	Right middle fossa, extending into the right orbit and suprasellar cistern	Oral prednisolone (for 2 months—no response) Subsequent interferon	Marked reduction with interferon treatment
Tortori et al., 1999 [14]	1 month, female	Nil	Right uncohippocampal	Observation	Resolution
Tortori et al., 1999 [14]	1.5 months, Female	Nil	Left CPA, leptomeningeal enhancement at cerebellar surface	Observation	Resolution
Tortori et al., 1999 [14]	18 months, Female	Nil at presentation, developed ataxia	Right CPA, hypothalamus	Systemic steroids, Endovascular treatment with contour particles	Unchanged following Steroids, Partial resolution following endovascular intervention
Tortori et al., 1999 [14]	1 month, female	Nil	Left CPA, persistent trigeminal artery	Systemic steroids	Lost to follow up
Poetke et al., 2002 [15]	10 weeks, male	Nil	CPA, leptomeningeal enhancement on cerebellar surface	Nil	Unknown
Le Bihannic et al., 2005 [16]	6 weeks, male	Vomiting, disturbance in consciousness, seizures	Anterior choroidal artery, right temporal lobe	Nil	Intracranial haemorrhage, death
Ersoy et al., 2005 [17]	8 months, female	Nil	Lateral medullary cistern, IAC, fourth ventricle	Oral prednisolone	Marked reduction in lesion size
Karikari et al., 2006 [18]	3 months, male	Central hypotonia	Fourth ventricle, left CPA	Surgical resection	Resolution
Judd et al., 2007 [19]	3 weeks, female	Nil	IAC/CPA	Oral prednisolone	Resolution
Judd et al., 2007 [19]	3 weeks, female	Right facial paresis	IAC/CPA, fourth ventricle	Oral prednisolone	Resolution
Judd et al., 2007 [19]	6 weeks, female	Nil	IAC/CPA, fourth ventricle	Intralesional triamcinolone	Resolution
Judd et al., 2007 [19]	8 weeks, female	Nil	IAC, Meckel’s cave, cavernous sinus	Intralesional triamcinolone	Resolution
Poindexter et al., 2007 [20]	2.5 months, female	Reduced truncal tone	Left IAC	Observation	Partial involution, developmental delay, diffuse hypotonia
Daenekindt et al., 2008 [21]	7 weeks, male	Enlarged head circumference	Right temporal fossa	Biopsy, Endovascular embolization, Surgical resection	Resolution
Frei-Jones et al., 2008 [22]	Newborn, female	Left CNVII palsy, left sensorineural hearing loss	Middle cranial fossa, temporal bone, posterior fossa	Biopsy, Thalidomide	Partial Resolution
Heyer et al., 2008 [23]	6 months, female	Nil	Left IAC	Observation	Unchanged
Uyama et al., 2008 [24]	4 months, female	Hydrocephalus	Left cerebellar hemisphere	Neuroendoscopic fenestration of cysts, Surgical resection of lesion	Resolution
Viswanathan et al., 2009 [2]	3 weeks, female	Hydrocephalus	Quad plate cistern, pineal region, left CPA	Corticosteroids	Reduction in lesion size
Viswanathan et al., 2009 [2]	9 weeks, female	Nil	Left cavernous sinus, Meckel’s cave, IAC	Corticosteroids	Lost to follow-up
Viswanathan et al., 2009 [2]	4 months, female	Hydrocephalus	Fourth ventricle, left IAC, CPA	Corticosteroids	Reduction in lesion size
Viswanathan et al., 2009 [2]	3 months, female	Left ptosis	Fourth ventricle, left foramen of Luschka, quad plate cistern	Interferon, OK432, Subsequent corticosteroids	Reduction in lesion size
Viswanathan et al., 2009 [2]	7 weeks, female	Right proptosis	Right temporal fossa, cavernous sinus, Meckel’s cave, sella, quad plate cisterns	Corticosteroids	Reduction in lesion size
Viswanathan et al., 2009 [2]	7 weeks, male	Nil	Fourth ventricle right foramen of Luschka, IAC	Nil	Reduction in lesion size
Viswanathan et al., 2009 [2]	3 weeks, male	Nil	Right CPA, foramen of Luschka, fourth ventricle	Corticosteroids, Interferon	Minimal response to corticosteroids, reduction in lesion size with Interferon
Viswanathan et al., 2009 [2]	Infancy, female	Nil	Peri-mesencephalic cistern, sella, cavernous sinus, left CPA	Interferon	Reduction in lesion size
Viswanathan et al., 2009 [2]	3 months, female	Nil at presentation, subsequent stroke and hydrocephalus	Right cavernous sinus, CPA	Corticosteroids	Reduction in lesion size
Philpott et al., 2012 [25]	12 months, female	Enlarged head circumference	Dura of right parietal lobe	Surgical resection	Resolution
Zheng et al., 2012 [26]	3 years, male	Somnolence, right CNIII palsy	Middle cranial fossa	Surgical resection	Resolution
Jalloh et al., 2014 [27]	2 weeks, Male	Tense anterior fontanelle, enlarging head circumference, seizures	Left middle cranial fossa	Biopsy, Surgical resection	Residual cyst, no recurrence
Benvenisti et al., 2014 [28]	4 weeks, female	Nil	Left posterior fossa	Oral propranolol	Reduction in lesion size, maintained at 12 months
Antonov et al., 2015 [29]	3 months, Female	Nil	Middle cranial fossa, right cavernous sinus, prepontine cistern, infratemporal fossa	Oral propranolol	Resolution
Antonov et al., 2015 [29]	3 weeks, female	Nil	Right lateral ventricular trigone	Oral propranolol	Resolution
El Rassi et al., 2015 [30]	5 weeks, female	Left CN V and VII palsy (PHACE syndrome)	Left CPA, IAC	Oral propranolol	Improvement in facial lesion, status of intracranial hemangioma not described
Cavalheiro et al., 2016 [31]	33 weeks gestation, male	Nil	Posterior fossa	Oral propranolol	Resolution
Kang et al., 2016 [8]	1 month, male	Nil	CPA	Oral propranolol	Resolution
Shakir et al., 2016 [32]	2 weeks, female	Hydrocephalus	Posterior fossa	Surgical resection	Resolution postoperative enlarging head circumference requiring VP shunt
Dalsin et al., 2016 [33]	37 weeks gestation, female	Diagnosed on antenatal ultrasound	Left middle cranial fossa	Surgical resection	Resolution, no neurological deficits
Haine et al., 2017 [34]	3 weeks, male	Symptoms of raised ICP	Posterior fossa	Surgical decompression oral prednisolone	Resolution on imaging
Friedland et al., 2017 [35]	1 week, male	Nil	Not specified	Observation	Spontaneous resolution
Naughton et al., 2020 [5]	6 weeks, female	Right CNVII palsy	Right orbit, right CPA and Meckel’s cave	Oral propranolol and topical timolol maleate 0.5%	Resolution
Dermesropian et al., 2021 [6]	33 weeks gestation, male	Diagnosed on antenatal ultrasound	Middle cranial fossa with extracranial infratemporal extension	Oral propranolol	Reduction in lesion size with mild left hemiparesis
Zamil et al., 2022 [7]	2 weeks, female	Right CNVII palsy	Dura of right temporal lobe	Oral propranolol	Resolution

In the analysis of the 36 cases done by Kang et al. in 2016, it was found that the cerebellopontine angle had been the most common location of involvement. Other locations included the internal auditory canal, middle and temporal cranial fossa and fourth ventricle. Their association with posterior fossa brain malformations, hemangiomas, arterial anomalies, coarctation of the aorta and cardiac defects, and eye abnormalities (PHACES) was found in nine of the cases [8]. Oral prednisolone was the most used pharmacological intervention in treatment and the mainstay of steroid therapy for this condition [5,9]. In 2008, Léauté-Labrèze et al. described the efficacy of oral propranolol in the management of infantile hemangioma as it is also well tolerated by the patients [5,10]. Surgical intervention is necessary in the presence of raised intracranial pressure or preoperative diagnosis is not confirmed and a more serious process is suspected [11]. Surgical resection is associated with high risk of bleeding as the vascular nature of the hemangioma which can accompanied by high morbidity and mortality [11]. In our case, surgical resection was done with minimal blood loss of 70cc without any complications; the patient's presenting symptoms markedly improved after surgery. 

Surgery has been performed as a treatment for these benign lesions in 10 reported cases as their rapid continuous growth had caused neurological complications. None of these cases had PHACES or additional hemangiomas. Others were managed pharmacologically with steroids, interferon therapy and thalidomide and some were watchfully observed. 

## Conclusions

Infantile hemangioma is a benign disease entity that is separate from cavernous hemangioma and hemangioblastoma, and it should be considered in the differential diagnosis of patients coming with intracranial lesion during infancy. Our report represents a very rare case of an isolated infantile hemangioma which presented in a rare location. We did an extensive literature review to review the previously reported cases and update the current knowledge regarding recent modalities in the management of such cases. Surgical resection is associated with high morbidity and pharmacological intervention should be considered first if possible with the exception of the conditions that necessitate urgent surgical intervention as the raised intracranial pressure from the compressive effect of the hemangioma or rapid neurological deterioration.
